# Induction of Labor in Late-Term and Post-Term Pregnancies Using Double-Balloon Catheter for Cervical Ripening: Predictors of Prolonged Labor Across Four Combined Augmentation Protocols in a Retrospective Cohort Study

**DOI:** 10.3390/jcm15052011

**Published:** 2026-03-05

**Authors:** Sadık Kükrer, Sefa Arlıer

**Affiliations:** Department of Obstetrics and Gynecology, University of Health Sciences, Adana City Training and Research Hospital, Mithat Ozsan Boulevard, Kısla District, 4522 Street No. 1, Adana 01140, Turkey; sefaarlier@gmail.com

**Keywords:** labor induction, post-term pregnancy, double-balloon catheter, cervical ripening, Bishop score, misoprostol, dinoprostone, oxytocin, uterine hyperstimulation

## Abstract

**Background/Objectives**: Induction of labor at ≥41 weeks with an unfavorable cervix is challenging. Comparative evidence for double-balloon catheter (DBC)-based augmentation protocols is limited. We aimed to estimate the frequency of prolonged labor, compare four DBC-based protocols, and identify predictors of timely vaginal delivery in the study population. **Methods**: This retrospective cohort study analyzed 709 women with singleton, cephalic pregnancies at ≥41 weeks and a Bishop score of ≤6 who achieved vaginal delivery following DBC-based induction at a tertiary referral center (2017–2026). The protocols comprised DBC alone or in combination with oxytocin, dinoprostone, or misoprostol. The primary outcome was vaginal delivery within 24 h of DBC insertion. Multivariable logistic regression and Kaplan–Meier analyses were performed, adjusting for maternal age, parity, body mass index, and post-ripening Bishop score changes. **Results**: Prolonged labor (≥24 h) occurred in 10.2% of vaginal deliveries and was associated with significantly elevated maternal infectious morbidity and adverse neonatal respiratory outcomes. The median induction-to-delivery interval was shortest with DBC plus misoprostol and longest with DBC plus dinoprostone (*p* < 0.001). Uterine hyperstimulation was most frequent with misoprostol (21.2% of cases). Post-ripening Bishop score change emerged as the strongest predictor of timely delivery (adjusted OR 4.72, 95% CI 2.99–7.43), whereas advancing maternal age reduced the odds of timely delivery (adjusted OR 0.65 per year, 95% CI 0.57–0.75). The prediction model demonstrated excellent discrimination (AUC = 0.924). **Conclusions**: In late-term and post-term DBC-based inductions, prolonged labor affected 10% of vaginal deliveries and substantially increased maternal and neonatal morbidity. DBC combined with misoprostol achieved the shortest delivery interval but carried the highest risk of hyperstimulation, whereas DBC combined with oxytocin offered the most favorable uterine activity profile. Post-ripening cervical reassessment, particularly changes in the Bishop score, enables evidence-based risk stratification and may guide the selection of individualized protocols.

## 1. Introduction

Labor induction has become a central component of contemporary obstetric practice, and its use has increased substantially in high-income health systems. Nationwide registry analyses have documented sustained growth in induction rates without a commensurate rise in cesarean deliveries, supporting the view that induction has become an increasingly routine pathway to birth [1]. This shift is particularly consequential in late-term (41 + 0 to 41 + 6 weeks) and post-term (42 + 0 weeks or later) pregnancies, when the balance between continued expectant management and delivery becomes increasingly time-sensitive. Individual participant data meta-analyses of randomized controlled trials indicate that induction at 41 weeks improves perinatal outcomes compared with expectant management until 42 weeks, without an associated increase in cesarean delivery [2].

Baseline cervical favorability is a key determinant of induction efficiency. When the cervix is unfavorable, cervical ripening with either mechanical or pharmacological methods is typically required before effective labor can be established [3]. Mechanical ripening with balloon catheters has been widely adopted in clinical practice and is supported by comparative studies. In an individual participant data meta-analysis, balloon catheters were associated with cesarean delivery rates comparable to those of vaginal prostaglandins, similar maternal safety outcomes, and fewer adverse perinatal events in the balloon catheter group [4]. Systematic reviews evaluating mechanical approaches [3], further support balloon catheter ripening as a core component of contemporary induction protocols.

Among the mechanical ripening options, double-balloon catheters (DBC) are particularly relevant in late-term and post-term pregnancies. The MAGPOP randomized trial, which compared silicone DBC with a slow-release dinoprostone system, demonstrated clinically meaningful differences in induction trajectories in this higher-risk gestational context [5]. In parallel, cohort studies comparing DBC with dinoprostone in multiparous women have reported broadly similar delivery efficacies but differences in selected intrapartum safety outcomes [6]. Collectively, these findings suggest that both the choice of ripening device and the subsequent augmentation and monitoring strategies can substantially influence the overall induction performance.

However, cervical ripening is seldom a stand-alone intervention. In contemporary practice, institutional protocols commonly use multistep pathways that augment labor during or after mechanical ripening, most often using oxytocin or prostaglandins. Evidence syntheses have demonstrated that combining mechanical and pharmacologic methods meaningfully shortens time-to-delivery. A network meta-analysis of induction strategies reported that combined approaches achieved higher rates of vaginal delivery within 24 h than single-modality regimens, while also highlighting substantial heterogeneity in protocol design across trials [7]. Randomized studies have further demonstrated that selected mechanical–pharmacologic combinations shorten the induction-to-delivery interval without increasing cesarean delivery rates in appropriately selected populations [8]. Safety considerations remain central to protocol selection. Systematic reviews and meta-analyses comparing misoprostol and dinoprostone generally report similar rates of major maternal and neonatal outcomes while emphasizing the need for careful surveillance for uterine hyperstimulation with prostaglandin-based regimens [9]. In addition, observational evaluations of DBC-based protocols, including those incorporating oxytocin augmentation, suggest that induction efficacy is influenced by multiple maternal and intrapartum factors, underscoring the value of prediction-focused analyses in real-world practice settings [10].

Despite the substantial literature on labor induction, critical gaps persist regarding DBC-based induction in late-term and post-term pregnancies. First, most comparative studies prioritize the mode of delivery, whereas prolonged labor is a distinct and clinically consequential outcome that substantially increases the burden of intrapartum interventions and hospital resource utilization. Second, comparative data evaluating multiple augmentation pathways within a unified DBC-based framework are severely limited. Third, the interplay among baseline cervical status, maternal characteristics, and augmentation strategies as determinants of prolonged labor in this setting remains poorly characterized.

In conclusion, we conducted a retrospective cohort study of late-term and post-term singleton cephalic pregnancies with unfavorable cervices undergoing DBC-based induction. Our objectives were to identify independent predictors of prolonged labor and to compare the induction efficiency and safety across four augmentation protocols: DBC alone, DBC plus oxytocin, DBC plus dinoprostone, and DBC plus misoprostol. We hypothesized that baseline cervical status and maternal parity would emerge as primary determinants of prolonged labor risk and that augmentation protocol selection would independently influence the probability of achieving vaginal delivery within 24 h. Clarifying these relationships has substantial implications for individualized protocol selection, with the direct capacity to reduce maternal and neonatal morbidity associated with prolonged labor and inform evidence-based clinical guidance.

## 2. Materials and Methods

### 2.1. Ethics Approval and Consent

This retrospective cohort study spanned 1 November 2017 to 1 January 2026 and was approved by the Institutional Ethics Committee (protocol no. 825; 23 October 2025). This study adhered rigorously to the principles of the Declaration of Helsinki. The retrospective design, which used routinely collected clinical data, obviated the need for individual informed consent under institutional policy and applicable regulatory frameworks. We implemented comprehensive data protection protocols: all patient information was de-identified before analysis, ensuring absolute confidentiality, consistent with institutional governance for secondary clinical data utilization. This manuscript fully adheres to the Strengthening the Reporting of Observational Studies in Epidemiology (STROBE) guidelines for cohort studies.

### 2.2. Study Population and Participant Selection

Participants were identified using the electronic medical record system of the Adana City Training and Research Hospital. A systematic retrospective analysis was conducted encompassing 1022 consecutive women with singleton live fetuses in cephalic presentation who underwent double-balloon catheter (DBC) cervical ripening for labor induction at ≥41 weeks and 0 days of gestation, spanning late-term (41 + 0 to 41 + 6 weeks) and post-term (≥42 + 0 weeks) pregnancies [11]. Each case mandated explicit documentation of obstetric indications for induction in the medical record.

Baseline cervical status at induction was assessed using the Bishop score, which rigorously integrates cervical dilation, effacement, fetal station, cervical consistency, and cervical position. The Bishop score provides a definitive characterization of initial cervical favorability, enabling robust risk-stratification analyses. For interpretive precision, scores ≤5 indicated unfavorable cervices, scores 6–7 indicate intermediate favorability, and scores ≥8 indicate favorable cervical status [7,12,13,14].

### 2.3. Exclusion Criteria

Women were excluded if they had multiple gestations, non-cephalic presentation, intrauterine fetal demise, major fetal anomalies, prior uterine surgery (cesarean delivery or myomectomy), active genital herpes infection, placenta previa or vasa previa, cervical cerclage in situ, gestational age <41 + 0 weeks, incomplete medical records, protocol deviations during induction, or maternal request for cesarean delivery.

Our ascertainment of outcomes from electronic clinical records spanning induction initiation through delivery obviated the need for post-discharge follow-up to establish the primary endpoint of prolonged labor in this cohort. We evaluated four DBC-based induction protocols: Group 1 (DBC alone), Group 2 (DBC plus oxytocin), Group 3 (DBC plus dinoprostone), and Group 4 (DBC plus misoprostol). The protocol assignment followed established clinical practices without randomization. Section 2.4 delineates the allocation methodology with precision.

#### Analytic Cohort Restriction

This analysis was restricted to women who achieved vaginal delivery (*n* = 709 of 1022 women; 69.4%). Women who underwent cesarean delivery (*n* = 313; 30.6%) were excluded from the primary time-to-delivery and prolonged labor analyses because the decision to proceed with cesarean delivery terminates the ongoing induction course and fundamentally precludes meaningful assessment of physiologic labor progression. In this methodological context, cesarean delivery constitutes a definitive intervention that irreversibly alters the temporal sequence of delivery and precludes valid incorporation into continuous time-to-delivery metrics intended to characterize the natural evolution of the induction-to-vaginal-delivery pathway.

### 2.4. Induction Procedures

A double-balloon catheter (Cook Medical, Bloomington, IN, USA) was inserted using a rigorous aseptic technique under direct visualization. The intrauterine balloon was inflated with precisely 40 mL of normal saline, and the vaginal balloon was inflated with exactly 80 mL [15,16]. The catheter remained in situ for up to 12 h or until spontaneous expulsion [15,16]. Cephalic presentation was confirmed by a comprehensive systematic clinical assessment immediately before double-balloon catheter insertion and was rigorously reassessed throughout the ripening period when clinically indicated, specifically to address suspected unstable lie or uncertainty regarding the presenting part of the fetus. Our meticulous surveillance protocol documented zero cases of fetal head displacement to the transverse or other non-vertex positions following catheter insertion in this cohort.

We implemented the following augmentation regimens with absolute fidelity to the protocol. Oxytocin: low-dose intravenous infusion initiated at precisely 2 mU/min with systematic upward titration by 2 mU/min increments every 20 min to a definitive maximum of 30 mU/min, administered via a calibrated infusion pump under continuous electronic fetal monitoring. Dinoprostone: Controlled-release 10 mg vaginal insert positioned in the posterior vaginal fornix with removal specified at 12 h or upon achievement of active labor. Misoprostol: 25 μg vaginal administration every 4 h for a maximum of 6 doses, with rigorous assessment for tachysystole before each subsequent dose [17,18].

Augmentation protocol selection following DBC placement was determined by established institutional practice algorithms integrated with the attending obstetrician’s clinical expertise, systematically grounded in comprehensively documented clinical parameters, including parity, membrane integrity status, and baseline cervical examination findings. The retrospective study design inherently precluded prospective protocol standardization; however, comprehensive institutional clinical practice guidelines established a robust, evidence-based framework that minimized arbitrary variation and ensured methodologically rigorous decision-making throughout the study period.

#### Timing and Sequence of Augmentation: Combined Mechanical–Pharmacological Protocol

Our institutional protocol implements a definitive combined (concomitant) methodology wherein mechanical cervical ripening via double-balloon catheter and pharmacologic augmentation are initiated simultaneously and maintained throughout the induction trajectory, strategically exploiting rigorously validated synergistic mechanisms that optimize biomechanical cervical remodeling while contemporaneously amplifying myometrial contractile responsiveness, as comprehensively demonstrated through contemporary high-impact randomized controlled trials and systematic meta-analyses [7,19,20]. This evidence-based concomitant approach constitutes a paradigmatic departure from conventional sequential protocols that defer pharmacologic agent administration exclusively until post-catheter removal, thereby forfeiting the demonstrable efficacy conferred by concurrent dual-mechanism cervical maturation and uterine preparation.

### 2.5. Data Sources and Measurement

We performed comprehensive retrospective data extraction from the institutional electronic medical record system and delivery registry using a rigorously validated, standardized abstraction instrument with pre-specified data elements and quality control checkpoints. Systematically abstracted variables encompassed comprehensive maternal demographics and baseline clinical parameters, including age, body mass index (BMI, calculated as weight in kilograms divided by height in meters squared), parity, and precisely documented gestational age at induction initiation prespecified obstetric indication(s) for induction with explicit clinical justification, the protocol-specific induction regimen assigned, comprehensive baseline cervical assessment metrics, including complete Bishop score components and transvaginal cervical length measurement, and the complete array of pre-specified maternal and neonatal outcomes defined a priori in accordance with established international standardized definitions.

Intrapartum blood loss was quantified using a combination of gravimetric measurements and standardized visual assessments. For vaginal deliveries, blood-soaked materials were weighed using calibrated scales, and the collected blood was measured using graduated containers. Postpartum hemorrhage was defined as an estimated blood loss of ≥500 mL following vaginal delivery or ≥1000 mL following cesarean delivery, in accordance with the American College of Obstetricians and Gynecologists (ACOG) criteria [4,15].

Uterine contractile abnormalities were classified according to the standardized National Institutes of Child Health and Human Development (NICHD) definitions. Uterine tachysystole was defined as >5 contractions in 10 min, averaged over 30 min. Uterine hyperstimulation was defined as tachysystole accompanied by fetal heart rate (FHR) abnormalities, including persistent late decelerations, prolonged decelerations, baseline fetal tachycardia, and decreased FHR variability. Uterine hypertonus was defined as a single contraction exceeding 2 min in duration. Uterine activity and FHR patterns were continuously monitored using cardiotocography throughout the induction period [3,4,21].

### 2.6. Cervical Assessment

The baseline cervical status at the start of induction was assessed using the Bishop score (cervical dilation, effacement, fetal station, cervical consistency, and cervical position). A baseline Bishop score of ≤6 was used to define an unfavorable cervix for cohort eligibility [22]. A modified Bishop score was calculated using a pre-specified institutional scoring schema (JCM_Supplementary_Contents, Table S2) [23,24,25,26]. The change in Bishop score was defined as the difference between the post-ripening and baseline Bishop assessments documented in the medical record [23,24,25,26].

### 2.7. Study Outcomes

The prespecified primary outcome was vaginal delivery within 24 h of double-balloon catheter insertion, operationally defined as an insertion-to-delivery interval of less than 24 h, a clinically meaningful threshold for induction efficiency and prolonged labor-associated morbidity. Prolonged labor, defined as an insertion-to-delivery interval of 24 h or longer, was the principal secondary outcome.

Additional prespecified secondary outcomes included cesarean delivery, induction-to-active-phase interval, total labor duration, uterine hyperstimulation [3], intrapartum fever [27], clinical chorioamnionitis [27], postpartum hemorrhage (>500 mL after vaginal delivery, >1000 mL after cesarean section) [28], severe perineal laceration (third- or fourth-degree) [29], blood transfusion requirement, and neonatal outcomes (5 min Apgar score <7, neonatal intensive care unit (NICU) admission [30], and umbilical arterial pH < 7.10) [31]. Respiratory distress was defined as the need for continuous positive airway pressure (CPAP) or endotracheal intubation.

### 2.8. Bias and Confounding

The retrospective design with a nonrandomized protocol assignment created an inherent confounding-by-indication risk. We implemented rigorous bias mitigation strategies by restricting analyses to women meeting prespecified eligibility criteria, systematically excluding records with missing data or intrapartum protocol deviations, and employing multivariable logistic regression with a priori adjustment for clinically essential covariates, including protocol assignment, maternal age, parity, body mass index, gestational age at induction, obstetric indication, baseline Bishop score, and post-ripening Bishop score change.

### 2.9. Study Size and Power Analysis

The prevalence of prolonged labor (≥24 h) in the vaginal delivery cohort was 10.2% (72/709 participants). The multivariable logistic regression model was specified with vaginal delivery within 24 h (<24 h) as the dependent variable (events: *n* = 637). Although the available event count did not constrain the number of candidate predictors, we prespecified a parsimonious set of clinically relevant variables to improve interpretability and reduce overfitting. With *n =* 709, the study had 80% power to detect odds ratios ≥1.7 in logistic regression (α = 0.05). For comparisons among the four protocols (average 177 participants per group), an 85% power was estimated for a medium effect size (Cohen’s W = 0.30). Internal validation used 1000 bootstrap replications to obtain optimism-corrected performance estimates.

### 2.10. Statistical Analysis

Statistical analyses were performed using Jamovi (version 2.6.44; The Jamovi Project, Sydney, Australia) and JASP (version 0.19.3; JASP Team, University of Amsterdam, Amsterdam, The Netherlands) software. Statistical significance was set at *p* < 0.05. Normality was assessed using the Shapiro–Wilk test. As continuous variables were not normally distributed, they were summarized as medians (min–max) and compared using the Mann–Whitney U test (two groups) or Kruskal–Wallis test (four protocol groups), as appropriate. Categorical variables are presented as *n* (%) and were compared using Pearson’s chi-square test. Fisher’s exact test (also known as the Fisher–Freeman–Halton test) was used when the expected cell counts were low.

The time to vaginal delivery following induction was evaluated using Kaplan–Meier methods within the vaginal delivery cohort, with administrative censoring at 24 h. Vaginal deliveries occurring within 24 h were coded as events, whereas those occurring after 24 h were treated as censored observations at 24 h. Survival curves were compared using the log-rank test, with Breslow (generalized Wilcoxon) and Tarone–Ware tests reported as supportive comparisons. Pairwise comparisons were adjusted using the Bonferroni correction. Multivariable logistic regression was used to identify the independent predictors of vaginal delivery within 24 h (<24 h). Prolonged labor (≥24 h) was treated as a complementary outcome, such that lower odds of delivery within 24 h indicated a higher likelihood of labor. Model discrimination was assessed using the area under the receiver operating characteristic curve (AUC), and internal validation was performed using 1000 bootstrap resamples to obtain the optimism-corrected performance estimates. Analyses were conducted using complete cases for the variables included in each model, and no imputations were performed.

## 3. Results

**Figure 1:**
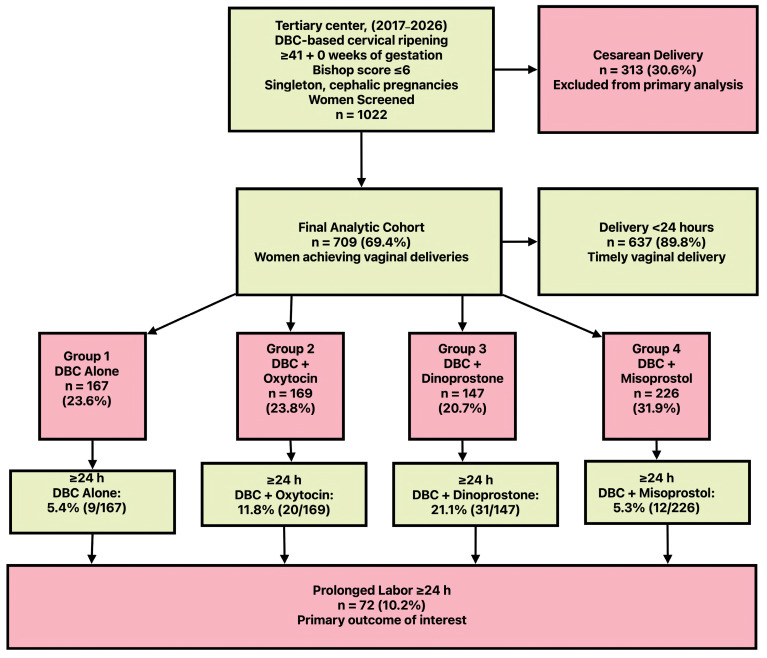
Study flow diagram with protocol-specific prolonged labor rates following DBC-based induction at ≥41 weeks.

Of 1022 consecutive women undergoing double-balloon catheter-based induction at ≥41 + 0 weeks gestation, 709 (69.4%) achieved vaginal delivery and constituted the definitive analytic cohort for comprehensive labor duration outcome assessment. Cesarean delivery occurred in 313 women (30.6%); the indications and associated maternal–neonatal outcomes for this subgroup were systematically characterized to establish a comprehensive procedural context. The institutional electronic medical record architecture, integrated with prospectively implemented standardized data collection protocols, ensured complete data for all variables incorporated into the primary analytic models, with no missing values documented across the entire study cohort (Figure 1).

Table 1 presents the baseline characteristics stratified by delivery timing. Women with prolonged labor (≥24 h) were older, had higher body mass index and parity, and were less frequently nulliparous than women delivering within 24 h (all *p* < 0.001). Baseline cervical favorability differed substantially; the prolonged labor group had lower admission Bishop scores (median 1.0 versus 4.0; *p* < 0.001), with all individual Bishop components showing significant differences. The distribution of induction protocols varied between the groups (*p* < 0.001), with dinoprostone-augmented protocols being more common in the prolonged labor group (43.1% vs. 18.2%). Gestational age, ultrasonographic parameters, and pregnancy complications were similar between the groups.

Table 2 presents the cervical ripening response and labor duration metrics stratified by delivery timing. Women who achieved timely delivery demonstrated a significantly greater post-ripening change in Bishop score (*p* < 0.001). Prolonged labor was associated with substantially longer intervals across all temporal metrics (all *p* < 0.001), with median insertion-to-delivery intervals more than double those in the prolonged labor cohort.

Table 3 presents the maternal and neonatal outcomes stratified by delivery time. Prolonged labor was associated with significantly elevated rates of infectious morbidity (intrapartum fever, chorioamnionitis), uterine complications (hyperstimulation, postpartum atony, suspected rupture), severe obstetric trauma (third- or fourth-degree perineal lacerations, postpartum hysterectomy), blood transfusion requirement, and neonatal respiratory distress (all *p* < 0.001).

Among the 313 women (30.6%) who underwent cesarean delivery, the most frequent indications were failure of labor progression, unsuccessful induction, and non-reassuring fetal heart rate patterns. The distribution of indications did not differ significantly across the protocol groups (JCM_Supplementary_Contents, Table S3). No cesarean deliveries were attributed to malpresentation developing after double-balloon catheter insertion, and no such cases were documented in medical records.

The comparative maternal complications in the DBC-alone subgroup are presented in Table 4. Among women who underwent DBC without pharmacological augmentation, prolonged labor was associated with substantially elevated rates of severe obstetric trauma, including third- and fourth-degree perineal tears (66.7% vs. 1.3%), uterine hyperstimulation (77.8% vs. 7.0%), postpartum atony (88.9% vs. 4.4%), and blood transfusion requirement (44.4% vs. 2.5%; all *p* < 0.001). Suspected uterine rupture occurred exclusively in the prolonged labor cohort (22.2% vs. 0.0%, *p* = 0.003).

The protocol-specific outcomes are presented in Table 5. Prolonged labor rates differed significantly across protocols (*p* < 0.001), with dinoprostone demonstrating the highest frequency (21.1%) and misoprostol the lowest (5.3%). The median induction-to-delivery interval was shortest with misoprostol (9.6 h) and longest with dinoprostone (14.7 h; *p* < 0.001). Uterine hyperstimulation occurred most frequently with misoprostol (21.2%) than with other protocols (5.3–10.8%, *p* < 0.001). The clinical chorioamnionitis rates varied significantly across the groups (*p* = 0.022), ranging from 4.7% (oxytocin) to 12.8% (misoprostol). Neonatal outcomes, including 5 min Apgar scores and NICU admission rates, showed no significant differences between the protocols.

**Figure 2:**
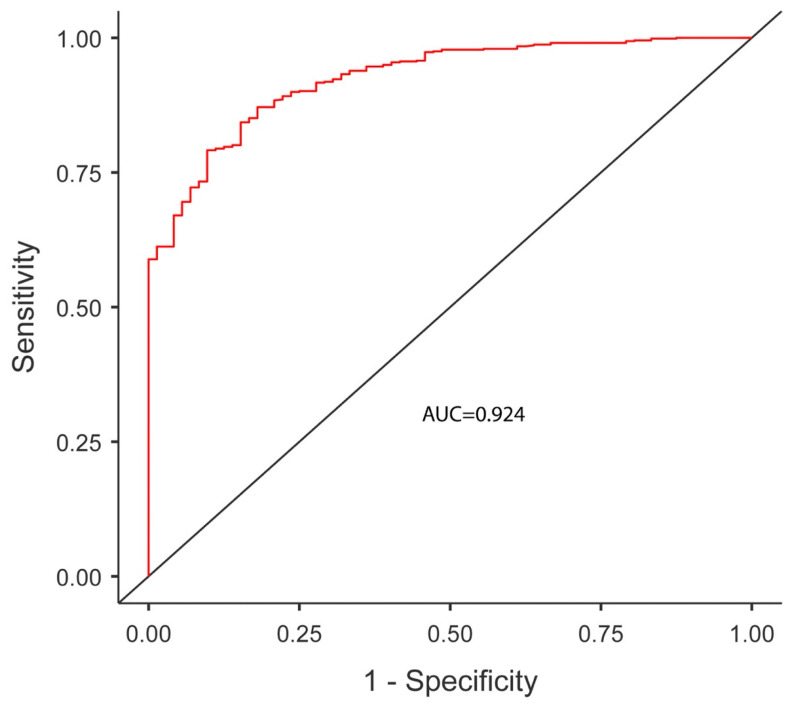
Receiver operating characteristic curve for the multivariable logistic regression model predicting vaginal delivery within 24 h (less than 24 h) following DBC-based induction.

The predictors of vaginal delivery within 24 h are presented in Table 6. In the multivariable analysis, change in Bishop score was the strongest predictor (adjusted OR 4.72, 95% CI 2.99–7.43, *p* < 0.001), followed by maternal age (adjusted OR 0.65, 95% CI 0.57–0.75, *p* < 0.001). Dinoprostone augmentation significantly reduced the odds of timely delivery (adjusted OR 0.08, 95% CI 0.03–0.22, *p* < 0.001). The model demonstrated excellent discrimination (AUC = 0.924) (Figure 2).

**Figure 3:**
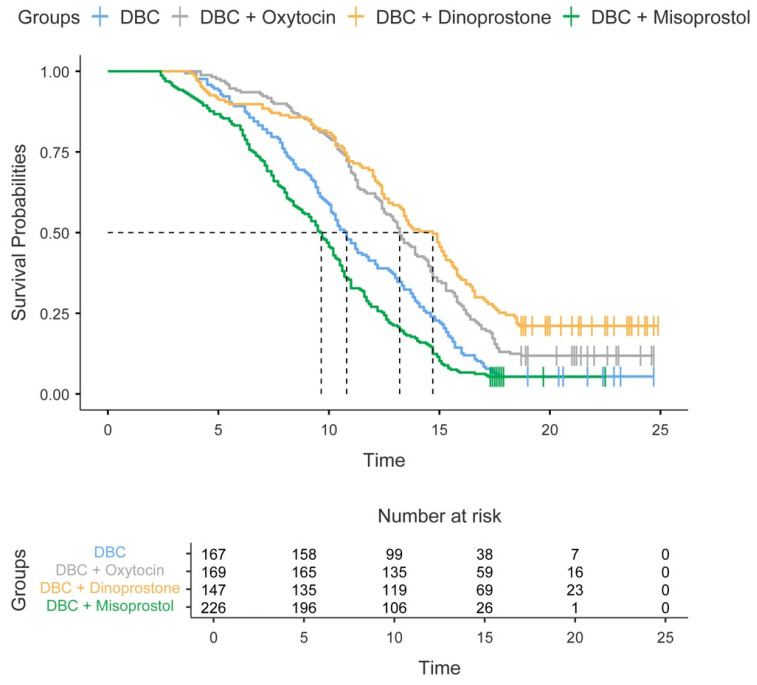
Kaplan–Meier curves for time to vaginal delivery by induction protocol. Survival probabilities represent the probability of remaining undelivered. Administrative censoring was applied after 24 h. The tick marks indicate the censored observations. The number at risk is shown below the plot.

The Kaplan–Meier time-to-delivery analysis stratified by protocol is presented in Table 7 and Figure 3. The median time to vaginal delivery was shortest with misoprostol (9.61 h) and longest with dinoprostone (14.7 h), with overall significant differences among the protocols (*p* < 0.001). Delivery within 24 h was achieved in 94.7%, 94.6%, 88.2%, and 78.9% of cases with misoprostol, DBC-alone, oxytocin, and dinoprostone, respectively. Pairwise comparisons demonstrated significant differences between dinoprostone and all other protocols (all *p* < 0.001).

## 4. Discussion

### 4.1. Principal Findings

In this retrospective cohort study of 709 post-term pregnancies undergoing induction with a double-balloon catheter (DBC), prolonged labor, defined as ≥24 h from DBC insertion, occurred in 10.2% of women who ultimately achieved vaginal birth. Notably, this duration threshold represents more than a simple time-based endpoint. It is associated with a clinically meaningful increase in both maternal and neonatal morbidity. The finding that post-ripening Bishop score change emerged as the strongest predictor of prolonged labor (AUC, 0.924) has direct clinical implications. Standardized cervical reassessment after ripening may enable evidence-based risk stratification and inform decisions regarding the earlier escalation of augmentation strategies or closer intrapartum surveillance. Protocol performance differed substantially across the four approaches: DBC combined with misoprostol demonstrated the most favorable time-to-delivery profile, whereas DBC combined with dinoprostone demonstrated the least efficiency.

### 4.2. The Relationship Between Cervical Length and Induction Success

Baseline cervical status clearly differentiated women with prolonged labor from those with timely courses. Those who experienced prolonged labor had a longer baseline transvaginal cervical length (38.0 vs. 30.0 mm) and markedly longer intervals from DBC insertion to the onset of active labor. These findings support a plausible mechanistic interpretation: pronounced cervical immaturity may allow mechanical ripening to occur, yet it delays the physiological transition into active labor, thereby prolonging subsequent stages of labor [32].

From a clinical perspective, transvaginal cervical length measurement may provide a pragmatic tool for risk stratification prior to induction. In our cohort, women with a baseline cervical length greater than 35 mm experienced extended insertion-to-active-labor intervals despite evidence of adequate mechanical ripening. This pattern suggests that when the cervix is markedly unripe, earlier pharmacologic augmentation may be more effective than prolonged mechanical ripening alone [32]. Collectively, these data support selecting an individualized protocol based on baseline cervical anatomy.

### 4.3. Clinical Implications of Prolonged Labor

The increased maternal morbidity observed in cases of prolonged labor likely reflects the multifactorial complexity of a protracted induction rather than a purely time-dependent effect. Longer labor duration increases the risk of ascending infection, elevates cumulative oxytocin exposure, and contributes to myometrial fatigue [33,34]. In addition, baseline characteristics that predispose women to prolonged labor, including an unfavorable cervix, nulliparity, and a higher body mass index, may independently increase the risk of intrapartum and postpartum complications [35,36,37]. Accordingly, these adverse outcomes should be interpreted as markers of a complex, higher-risk induction trajectory rather than as consequences attributable solely to elapsed time.

It is also important to recognize that post-term induction occurs in a clinical context with a higher baseline risk profile than that of spontaneous labor [2]. Contemporary evidence indicates that induction at 41 weeks reduces perinatal mortality and severe neonatal morbidity compared with expectant management, supporting a proactive approach to post-term pregnancy while acknowledging the operational and physiological complexities of induction [7]. In this setting, achieving delivery within 24 h represents both a meaningful clinical quality benchmark and a patient-centered outcome, with potential benefits including reduced hospital resource utilization and improved maternal satisfaction.

### 4.4. Protocol-Specific Efficacy–Safety Trade-Offs

Our results support an evidence-based approach to protocol selection. Among women with relatively favorable baseline cervical status, in whom efficiency is a priority, DBC plus misoprostol provides the shortest time-to-delivery when uterine activity is closely monitored [7,8]. The higher rate of uterine tachysystole or hyperstimulation (21.2%) should be interpreted in the context of post-term induction physiology rather than as an intrinsic marker of protocol toxicity [38]. Prostaglandin augmentation can accelerate both cervical ripening and myometrial contractility, thereby achieving a shorter median time-to-delivery (9.6 h) at the cost of an increased likelihood of heightened uterine activity, which warrants enhanced surveillance. In this sense, clinical decisions reflect a risk–benefit trade-off rather than a simple safety signal.

In contrast, in women with an unfavorable cervix or contraindications to prostaglandins, DBC plus oxytocin offered a more favorable uterine activity profile (5.3% hyperstimulation) with intermediate efficiency [19,20]. The longer delivery intervals observed with DBC plus dinoprostone may be partially explained by confounding by indication, as dinoprostone may have been preferentially used in patients with less favorable baseline cervical conditions. Consistent with this interpretation, the apparent protocol-specific differences diminished after multivariate adjustment, suggesting that baseline patient characteristics substantially shaped the observed protocol performance.

### 4.5. Prediction Model and Correct Interpretation of Adjusted Effects

In our logistic regression analysis, the outcome was vaginal delivery within 24 h. Accordingly, adjusted odds ratios below 1.0 indicate a lower likelihood of timely vaginal delivery and a greater probability of prolonged labor. The change in Bishop score emerged as the most robust independent predictor, supporting the concept that the early cervical response to ripening largely determines the subsequent induction trajectory. This interpretation is consistent with the broader evidence, indicating that both baseline cervical favorability and the magnitude of early cervical change are key determinants of induction success [32,37]. Prior reports similarly show that a shorter transvaginal cervical length is associated with more efficient physiological progression and a higher likelihood of successful induction [32,37]. Taken together, these data reinforce the clinical value of integrating baseline cervical assessment with early post-ripening response when stratifying the risk of women undergoing DBC-based induction.

### 4.6. Strengths and Limitations

The key strengths of this study include the large post-term cohort, protocol-stratified reporting of outcomes, and complementary analytic approaches, namely multivariable logistic regression and time-to-event methods, which together characterize both dichotomous endpoints and the full time-to-delivery distribution. The prediction model demonstrated excellent discriminatory performance (AUC 0.924) with acceptable calibration, supporting its potential clinical utility.

This study has several limitations. First, the retrospective, single-center design limited causal inference and increased the risk of residual confounding. Although we applied strict eligibility criteria and adjusted for confounding by indication, residual confounding cannot be fully eliminated in observational analyses. In addition, the primary analyses were restricted to women who achieved vaginal delivery, which may have introduced selection bias because vaginal birth is influenced by both baseline characteristics and intrapartum events. Future studies should evaluate the time-to-delivery in the entire induced cohort using methods that account for cesarean delivery as a competing outcome.

## 5. Conclusions

In late-term and post-term pregnancies undergoing induction with a double-balloon catheter, prolonged labor, defined as 24 h or longer, occurred in 10% of vaginal deliveries and was associated with substantially increased maternal and neonatal morbidity. Change in Bishop score was the strongest predictor of timely vaginal delivery, supporting routine post-ripening cervical reassessment as a pragmatic tool for risk stratification and intrapartum decision-making.

Protocol selection requires clinically meaningful trade-offs between efficiency and safety. DBC plus misoprostol was associated with the shortest time-to-delivery but necessitates enhanced uterine activity monitoring. In contrast, DBC plus oxytocin offered a more favorable uterine activity profile with intermediate efficiency. In contrast, DBC plus dinoprostone showed the least favorable performance in this cohort. Collectively, these findings support an individualized approach that integrates baseline cervical status with institutional monitoring capacity and escalation pathways. External validation and prospective comparative studies are warranted to refine evidence-based induction strategies for late-term and post-term pregnancies.

## Figures and Tables

**Table 1:** Baseline maternal, obstetric, and cervical characteristics of women who achieved vaginal delivery after DBC-based induction, stratified by induction-to-delivery interval.

*Characteristic*	≥24 h (*n* = 72)	<24 h (*n* = 637)	*p*-Value
***Protocol group distribution, n (%)***			
**DBC alone**	9 (12.5)	158 (24.8)	<0.001 *
**DBC + Oxytocin**	20 (27.8)	149 (23.4)	
**DBC + Dinoprostone**	31 (43.1)	116 (18.2)	
**DBC + Misoprostol**	12 (16.7)	214 (33.6)	
***Maternal demographic and obstetric characteristics***			
**Maternal age (years)**	35.0 [20.0–40.0]	29.0 [20.0–40.0]	<0.001 **
**Maternal height (cm)**	177.0 [165.0–184.0]	166.0 [155.0–185.0]	<0.001 **
**Maternal weight (kg)**	105.0 [88.0–117.0]	84.0 [50.0–120.0]	<0.001 **
**Maternal BMI at IOL (kg/m^2^)**	33.4 [30.7–35.0]	30.1 [20.8–35.4]	<0.001 **
**Gestational age at delivery**			**NS**
Late-term (41 + 0 to 41 + 6 weeks), *n* (%)	54 (75.0)	506 (79.4)	
Post-term (≥42 + 0 weeks), *n* (%)	18 (25.0)	131 (20.6)	
**Gravidity**	3.0 [1.0–8.0]	2.0 [1.0–8.0]	<0.001 **
**Parity**	2.0 [0.0–6.0]	1.0 [0.0–6.0]	<0.001 **
**Nulliparity, *n* (%)**	9 (12.5)	297 (46.6)	<0.001 *
***Baseline cervical assessment (standard Bishop score)***			
**Cervical dilatation, *n* (%)**			<0.001 *
0 cm (0 point)	72 (100.0)	333 (52.3)	
1–2 cm (+1 point)	0 (0.0)	107 (16.8)	
3–4 cm (+2 points)	0 (0.0)	101 (15.9)	
≥5 cm (+3 points)	0 (0.0)	96 (15.1)	
**Cervical position, *n* (%)**			<0.001 *
Posterior (0 point)	10 (13.9)	357 (56.0)	
Mid-position (+1 point)	62 (86.1)	194 (30.5)	
Anterior (+2 points)	0 (0.0)	86 (13.5)	
**Cervical consistency, *n* (%)**			<0.001 *
Firm (0 point)	72 (100.0)	239 (37.5)	
Moderately firm (+1 point)	0 (0.0)	297 (46.6)	
Soft (+2 points)	0 (0.0)	101 (15.9)	
**Cervical effacement (%)**	30.0 [0.0–30.0]	40.0 [0.0–80.0]	<0.001 **
**Fetal station, *n* (%)**			<0.001 *
−3 (0 point)	72 (100.0)	234 (36.7)	
−2 (+1 point)	0 (0.0)	260 (40.8)	
−1 (+2 points)	0 (0.0)	79 (12.4)	
0 (+3 points)	0 (0.0)	64 (10.0)	
**Bishop score upon admission**	1.0 [0.0–1.0]	4.0 [1.0–9.0]	<0.001 **
***Modified Bishop score parameters***			
**Modified cervical dilatation, *n* (%)**			<0.001 *
0 cm (0 point)	72 (100.0)	333 (52.3)	
1–2 cm (+2 points)	0 (0.0)	107 (16.8)	
3–4 cm (+4 points)	0 (0.0)	101 (15.9)	
>4 cm (+6 points)	0 (0.0)	96 (15.1)	
**Digital cervical length (modified Bishop), *n* (%)**			<0.001 *
3 cm (0 point)	72 (100.0)	68 (10.7)	
2 cm (+1 point)	0 (0.0)	55 (8.6)	
1 cm (+2 points)	0 (0.0)	190 (29.8)	
0 cm (+3 points)	0 (0.0)	324 (50.9)	
**Modified Bishop Score**	2.0 [−2.0–7.0]	3.0 [−2.0–11.0]	<0.001 **
**Transvaginal cervical length (mm)**	38.0 [31.0–46.0]	30.0 [4.0–46.0]	<0.001 **
***Ultrasonographic and clinical parameters***			
**Meconium-stained amniotic fluid, *n* (%)**	6 (8.3)	51 (8.0)	NS
**Amniotic Fluid Index (cm)**	12.2 [5.0–25.9]	13.0 [5.0–26.8]	NS
**Single Deepest Pocket (cm)**	6.0 [2.5–7.5]	6.1 [2.5–7.7]	NS
**Estimated Fetal Weight (grams)**	3340.0 [2500.0–4000.0]	3330.0 [2500.0–4000.0]	NS
**Fetal Biophysical Profile Score**	9.0 [6.0–10.0]	9.0 [6.0–10.0]	NS
**Oligohydramnios, *n* (%)**	15 (20.8)	161 (25.3)	NS
*Pregnancy complications*			
**Hypertensive disorder, *n* (%)**	9 (12.5)	74 (11.6)	NS
**Gestational Diabetes Mellitus, *n* (%)**	9 (12.5)	146 (22.9)	NS
**Fetal growth classification, *n* (%)**			NS
Small for gestational age (<10th percentile)	6 (8.3)	39 (6.1)	
Appropriate for gestational age (10th–90th)	62 (86.1)	559 (87.8)	
Large for gestational age (>90th percentile)	4 (5.6)	39 (6.1)	

Data are presented as *n* (%) or median [range]. * Chi-square or Fisher’s exact test. ** Mann–Whitney U test. DBC, double-balloon catheter; IOL, induction of labor; BMI, body mass index; NS, not significant (*p* ≥ 0.05). Bold *p*-values indicate statistical significance (*p* ≤ 0.05).

**Table 2:** Cervical ripening response and labor duration metrics stratified by induction-to-delivery interval following DBC-based induction.

*Characteristic*	≥24 h (*n* = 72)	<24 h (*n* = 637)	*p*-Value
***Cervical ripening response***			
Changes in cervical Bishop score after IOL	4.0 [2.0–4.0]	5.0 [2.0–10.0]	<0.001 **
***Time from PROM to IOL***			
No PROM, *n* (%)	47 (65.3)	305 (47.9)	0.003 *
≤12 h, *n* (%)	23 (31.9)	234 (36.7)	
>12 h, *n* (%)	2 (2.8)	98 (15.4)	
***Induction-to-labor intervals***			
Insertion to active labor (hours)	14.3 [11.2–16.0]	7.1 [1.1–13.4]	<0.001 **
Insertion to delivery (hours)	21.4 [17.3–24.9]	10.9 [2.4–18.7]	<0.001 **
***Labor stage durations***			
Length of first stage of labor (hours)	11.4 [10.4–19.8]	6.1 [1.0–10.9]	<0.001 **
Length of second stage of labor (minutes)	57.0 [51.0–70.0]	30.0 [5.0–54.0]	<0.001 **
Length of third stage of labor (minutes)	25.0 [20.0–30.0]	9.0 [1.0–23.0]	<0.001 **
Length of total labor (hours)	31.7 [24.1–39.9]	14.4 [4.6–24.8]	<0.001 **

Data are presented as *n* (%) or median [range]. * Chi-square or Fisher’s exact test. ** Mann–Whitney U test. IOL, induction of labor; PROM, premature rupture of membranes. Changes in Bishop score: post-ripening minus baseline. All time intervals were measured from the time of catheterization. Bold *p*-values indicate statistical significance (*p* ≤ 0.05).

**Table 3:** Intrapartum complications and maternal and neonatal outcomes among women who achieved vaginal delivery after DBC-based induction, stratified by induction-to-vaginal delivery interval (<24 h vs. ≥24 h).

*Characteristics*	≥24 h	<24 h	*p*-Value
	*(n = 72)*	*(n = 637)*	
***Maternal Characteristics***			
**Oxytocin augmentation, presence**	23 (31.9)	202 (31.7)	NS
**Artificial rupture of membranes (amniotomy), presence**	37 (51.4)	230 (36.1)	0.016 *
**Maternal Prepartum Hb levels (g/dL)**	12.0 [10.3–13.5]	11.9 [10.1–13.7]	**NS**
**Maternal Postpartum Hb (g/dL)**	10.4 [7.4–12.9]	11.0 [6.8–13.0]	0.001 **
**Maternal prepartum to postpartum hemoglobin difference (g/dL)**	0.8 [0.6–3.7]	0.8 [0.6–3.8]	<0.001 **
**Maternal Intensive Care Unit (MICU) admission, presence**	5 (6.9)	7 (1.1)	0.004 *
**Total maternal hospital stays (days)**	4.0 [2.8–5.2]	3.9 [2.8–5.2]	NS
***Maternal complications***			
**Intrapartum fever, presence**	18 (25.0)	46 (7.2)	<0.001 *
**Clinical chorioamnionitis, presence**	16 (22.2)	44 (6.9)	<0.001 *
**Precipitate labor, presence**	0 (0.0)	61 (9.6)	0.012 *
**Episiotomy plus vacuum extraction, presence**	26 (36.1)	90 (14.1)	<0.001 *
**Third- or fourth-degree perineal tears, presence**	7 (9.7)	25 (3.9)	0.035 *
**Internal Obstetric Anal Sphincter Injury (IOASI), presence**	3 (4.2)	14 (2.2)	NS
**Uterine suspected rupture, presence**	5 (6.9)	1 (0.2)	<0.001 *
**Uterine hyperstimulation, presence**	22 (30.6)	64 (10.0)	<0.001 *
**Postpartum uterine atony, presence**	27 (37.5)	30 (4.7)	<0.001 *
**Blood transfusions, presence**	21 (29.2)	19 (3.0)	<0.001 *
**Postpartum blood loss (mL)**	945.0 [670.0–2460.0]	830.0 [640.0–2470.0]	<0.001 **
**Postpartum Hysterectomy, presence**	3 (4.2)	3 (0.5)	0.016 *
***Neonatal characteristics and complications***			
**Birth weight (grams)**	3256.0 [2438.0–3900.0]	3246.0 [2438.0–3900.0]	NS
**Apgar Score 5th minute**			
**<7**	11 (15.3)	23 (3.6)	<0.001 *
**≥7**	61 (84.7)	614 (96.4)	
**Umbilical artery pH**	7.3 [7.1–7.4]	7.3 [7.0–7.4]	NS
**Respiratory distress requiring CPAP or intubation, presence**	13 (18.1)	35 (5.5)	<0.001 *

Data presented as *n* (%) or median [range]. * Chi-square or Fisher’s exact test. ** Mann–Whitney U test. Hb, hemoglobin; MICU, maternal intensive care unit; IOASI, internal obstetric anal sphincter injury; CPAP, continuous positive airway pressure; NS, not significant (*p* ≥ 0.05). Bold *p*-values indicate statistical significance (*p* ≤ 0.05).

**Table 4:** Comparative analysis of maternal complications associated with prolonged labor in the DBC-alone subgroup.

*Variables*	≥24 h (*n* = 9)	<24 h (*n* = 158)	*p*-Values
**Third- or fourth-degree perineal tears, presence**	6 (66.7)	2 (1.3)	<0.001 *
**Severe (OASI), presence**	2 (22.2)	2 (1.3)	0.015 *
**Uterine suspected rupture, presence**	2 (22.2)	0 (0.0)	0.003 *
**Uterine hyperstimulation, presence**	7 (77.8)	11 (7.0)	<0.001 *
**Postpartum atony, presence**	8 (88.9)	7 (4.4)	<0.001 *
**Blood transfusion, presence**	4 (44.4)	4 (2.5)	<0.001 *
**Postpartum blood loss *(mL)***	970.0 [710.0–2400.0]	800.0 [680.0–2470.0]	<0.001 **
**Postpartum hysterectomy, presence**	1 (11.1)	0 (0.0)	NS

Data presented as *n* (%) or median [range]. * Chi-square or Fisher’s exact test. ** Mann–Whitney U test. OASI, obstetric anal sphincter injury; NS, not significant (*p* ≥ 0.05). Analysis restricted to the DBC-alone group (no pharmacological augmentation). Bold *p*-values indicate statistical significance (*p* ≤ 0.05).

**Table 5:** Prolonged labor, delivery timing, and maternal and neonatal outcomes of augmentation protocol following DBC-based induction (vaginal delivery cohort).

*Outcomes*	DBC	DBC + Oxytocin	DBC + Dinoprostone	DBC + Misoprostol	*p*-Value
	*(n = 167)*	*(n = 169)*	*(n = 147)*	*(n = 226)*	
***Primary Outcome***					
Prolonged labor ≥24 h	9 (5.4)	20 (11.8)	31 (21.1)	12 (5.3)	<0.001 *
***Secondary Outcomes***					
Induction-to-delivery interval *(hours)*	10.8 [3.5–24.7]	13.2 [4.2–24.7]	14.7 [3.7–24.9]	9.6 [2.4–22.5]	<0.001 **
Vaginal delivery within 24 h (<24 h)	158 (94.6)	149 (88.2)	116 (78.9)	214 (94.7)	<0.001 *
***Maternal Complications***					
Uterine hyperstimulation	18 (10.8)	9 (5.3)	11 (7.5)	48 (21.2)	<0.001 *
Clinical chorioamnionitis	14 (8.4)	8 (4.7)	9 (6.1)	29 (12.8)	0.022 *
Postpartum blood loss (mL) †	810 [680–2470]	850 [640–2450]	850 [710–2460]	860 [670–2470]	<0.001 **
Third- or fourth-degree perineal tears	8 (4.8)	5 (3.0)	7 (4.8)	12 (5.3)	NS
***Neonatal Outcomes***					
5 min Apgar score <7	8 (4.8)	8 (4.7)	6 (4.1)	12 (5.3)	NS
NICU admission	9 (5.4)	8 (4.7)	12 (8.2)	19 (8.4)	NS
Umbilical arterial pH ‡	7.27 [7.11–7.35]	7.27 [7.14–7.34]	7.32 [7.10–7.39]	7.29 [7.01–7.35]	<0.001 **

Data are presented as *n* (%) for categorical variables and median [range] for continuous variables. * Pearson’s chi-square test or Fisher’s exact test (Fisher–Freeman–Halton) for categorical variables. ** Mann–Whitney U test for continuous variables. † Kruskal–Wallis test for comparison across the four protocol groups. ‡ Umbilical arterial pH was measured immediately after delivery. NICU, neonatal intensive care unit; NS, not significant (*p* ≥ 0.05). The analysis was restricted to women who achieved vaginal delivery (*n* = 709 of 1022 women who underwent induction; 69.4%). Bold *p*-values indicate statistical significance (*p* < 0.05).

**Table 6:** Univariable and multivariable logistic regression analyses of predictors of vaginal delivery within 24 h of DBC-based induction.

*Variables*	Univariable Analysis		Multivariable Analysis	
	OR (95% CI)	*p*-Value	aOR (95% CI)	*p*-Value
***Induction Protocol***				
DBC *(reference)*	1	-	1	-
DBC + Oxytocin	0.42 (0.18–0.94)	**0.04**	0.24 (0.09–0.64)	**0.004**
DBC + Dinoprostone	0.21 (0.09–0.45)	**<0.001**	0.08 (0.03–0.22)	**<0.001**
DBC + Misoprostol	1.02 (0.41–2.46)	0.972	0.56 (0.20–1.56)	NS
**Maternal age *(years)***	0.82 (0.77–0.86)	**<0.001**	0.65 (0.57–0.75)	**<0.001**
**BMI *(kg/m*^2^*)***	0.73 (0.65–0.80)	**<0.001**	1.26 (0.98–1.61)	NS
**Nulliparity, *presence***	6.11 (3.14–13.38)	**<0.001**	0.34 (0.10–1.22)	NS
**Bishop Score Changes**	2.08 (1.73–2.54)	**<0.001**	4.72 (2.99–7.43)	**<0.001**
**Cervical length *(mm)***	0.86 (0.83–0.90)	**<0.001**	-	- †
**PROM, *presence***	2.05 (1.24–3.45)	**0.006**	-	- †

The outcome was vaginal delivery within 24 h of DBC insertion. OR/aOR < 1 indicated lower odds of delivery within 24 h. OR: odds ratio; aOR: adjusted odds ratio; CI: confidence interval; BMI: body mass index; PROM: premature rupture of membranes; DBC: double-balloon catheter. † Variables removed during backward stepwise selection (*p* > 0.10). Bold *p*-values indicate statistical significance (*p* ≤ 0.05). *p*-values greater than or equal to 0.05 with “NS” (not significant).

**Table 7:** Kaplan–Meier analysis of time to vaginal delivery within 24 h by induction protocol (administrative censoring at 24 h).

*Variables*	DBC	DBC + Oxytocin	DBC + Dinoprostone	DBC + Misoprostol	*p*-Value
**Number at risk**	167	169	147	226	
**Events (delivery within 24 h)**	158	149	116	214	
**Censored at 24 h (delivery >24 h), *n* (%)**	9 (5.4)	20 (11.8)	31 (21.1)	12 (5.3)	
***Overall comparison (Log-rank, χ^2^ = 53.9, df = 3)***					**<0.001**
Median time to delivery (hours)	10.8	13.2	14.7	9.61	
95% CI	10.10–11.80	12.50–14.40	13.30–15.90	8.90–10.20	
Delivery within 24 h, *n* (%)	158 (94.6)	149 (88.2)	116 (78.9)	214 (94.7)	
***Quartiles (hours)***					
25th percentile	8.47	10.47	11.56	7.37	
50th percentile (median)	10.8	13.2	14.7	9.61	
75th percentile	13.76	17.63	19.18	12.81	
Restricted mean time to delivery up to 24 h	11.8	14.1	15.5	10.2	
SE	0.47	0.64	0.53	0.35	
***Pairwise comparisons (Log-rank, Bonferroni-adjusted)***					
vs. DBC	Reference	**0.001**	**<0.001**	NS	
vs. DBC + Oxytocin	-	Reference	NS	**<0.001**	
vs. DBC + Dinoprostone	-	-	Reference	**<0.001**	

Time-to-event analyses were performed using the Kaplan–Meier method with administrative censoring at 24 h. Overall comparisons: log-rank test. Pairwise comparisons: log-rank test with Bonferroni correction. CI, confidence interval; SE, standard error; NS, not significant (*p* ≥ 0.05). Bold *p*-values indicate statistical significance (*p* ≤ 0.05).

## Data Availability

De-identified patient data supporting this study were extracted from institutional electronic medical records. The datasets are not publicly available owing to patient privacy and institutional restrictions. Data may be provided by the corresponding author upon reasonable request, contingent on approval from the Institutional Ethics Committee and completion of any required institutional data access procedures.

## Supplementary Materials

The following supporting information can be downloaded from https://www.mdpi.com/article/10.3390/jcm15052011/s1, Complete survival statistics are provided in the Supplementary File JCM, Supplementary Content.docx. Table S1: The conventional Bishop score rubric was used for baseline cervical assessment before the IOL; Table S2: Modified Bishop’s Score [39].

## Abbreviations

AFI	Amniotic fluid index
AGA	Appropriate for gestational age
aOR	Adjusted odds ratio
AUC	Area under the curve
BMI	Body mass index
BPP	Biophysical profile
CI	Confidence interval
CPAP	Continuous positive airway pressure
DBC	Double-balloon catheter
EFW	Estimated fetal weight
GDM	Gestational diabetes mellitus
Hb	Hemoglobin
IOASI	Internal obstetric anal sphincter injury
IOL	Induction of labor
LGA	Large for gestational age
MICU	Maternal Intensive Care Unit
NICU	Neonatal Intensive Care Unit
OASI	Obstetric anal sphincter injury
OR	Odds ratio
PPH	Postpartum hemorrhage
PROMs	Premature rupture of membranes
ROC	Receiver operating characteristic
SDP	Single deepest pocket
SE	Standard error
SGA	Small for gestational age
STROBE	Strengthening the Reporting of Observational Studies in Epidemiology
SWEPIS	Swedish Post-term Induction Study
USG	Ultrasonography
