# Supplementary material for: Induction of Labor in Late-Term and Post-Term Pregnancies Using Double-Balloon Catheter for Cervical Ripening: Predictors of Prolonged Labor Across Four Combined Augmentation Protocols in a Retrospective Cohort Study
Source: J Clin Med. 2026 Mar 5;15(5):2011. doi: 10.3390/jcm15052011 (PMC12985892; doi:10.3390/jcm15052011)
Supplement: Supplementary file 1 [file jcm-15-02011-s001.zip › jcm-4147074-supplementary.pdf]

Journal of
Clinical Medicine

MDPI

Article

Supplementary Content

Methods

Table S1. The conventional Bishop score rubric was used for baseline cervical assessment before the IOL.

Parameter	0 Points	1 Point	2 Points	3 Points
Dilation (cm)	Closed	1–2	3–4	≥5
Effacement (%)	0–30%	40–50%	60–70%	≥80%
Station (fetal head)	-3	-2	−1 or 0	+1 or +2
Consistency	Firm	Moderately firm	Soft	—
Position	Posterior	Mid-position	Anterior	—

The Bishop score was calculated as the sum of five cervical examination components (dilation, effacement, fetal station, consistency, and position), yielding a total score ranging from 0 to 13; higher scores indicated a more favorable cervix. The fetal station is defined relative to the maternal ischial spine. Score interpretation: ≤5 suggests an unfavorable cervix, 6–7 is indeterminate, and ≥8 suggests a favorable cervix with a higher likelihood of vaginal delivery [1].

Table S2. Modified Bishop's Score.

Parameter	-1 Points	0 Points	1 Point	2 Points	3 Points	4 Points	6 Points
**Cervical dilation (cm)**	–	Closed	–	1–2	–	3–4	>4
**Cervical length (cm)**	–	3	2	1	0	–	–
**Station (fetal head)**	–	-3	-2	−1 or 0	+1 or +2	–	–
**Cervical Position**	–	Posterior	Mid-position	Anterior	–	–	–
**Consistency**	–	Firm	Moderately firm	Soft	–	–	–
**Number of Prior Vaginal Deliveries (Norm: 0–10)**							
**Pre-eclampsia**	–	No	Yes		–	–	–
**Postdate pregnancy**	Yes	No	–	–	–	–	–
**Nulliparity**	Yes	No	–	–	–	–	–
**Premature Preterm Rupture of Membranes (PPROM)**	Yes	No	–	–	–	–	–

The modified Bishop score was calculated as the sum of five cervical examination components (dilation, effacement, fetal station, consistency, and position), yielding a total score ranging from 0 to 13; higher scores indicate a more favorable cervix. The fetal station is defined relative to the maternal ischial spine. Score interpretation: ≤5 suggests an unfavorable cervix; 6–7 is indeterminate; and ≥8 suggests a favorable cervix with a higher likelihood of vaginal delivery.

In this cohort, **Table S1** presents the exact scoring rubric used for the conventional Bishop score at admission and immediately before the initiation of the induction protocol,

ensuring a transparent and reproducible classification of the baseline cervical status. An unfavorable cervix was operationalized using the low Bishop score thresholds commonly used in induction research and clinical decision-making [2,3].

Table S2 presents the institutionally modified cervical favorability scoring rubric used to standardize the baseline assessment in retrospective records and to incorporate structured cervical elements, alongside objective measures such as transvaginal cervical length. This approach is supported by evidence that the clinical Bishop score is subjective and poorly reproducible. In contrast, ultrasound-based parameters analogous to Bishop components show good-to-excellent repeatability and correlate well with the corresponding clinical domains [2,3].

References

1. Levine, L.D.; Downes, K.L.; Parry, S.; Elovitz, M.A.; Sammel, M.D.; Srinivas, S.K. A validated calculator to estimate risk of cesarean after an induction of labor with an unfavorable cervix. *Am J Obstet Gynecol* **2018**, *218*, 254 e251–254 e257, doi:10.1016/j.ajog.2017.11.603.
2. Bas Lando, M.; Majida, E.; Solnica, A.; Helman, S.; Margaliot Kalifa, T.; Grisaru-Granovsky, S.; Reichman, O. Nulliparas at Term with Premature Rupture of Membranes and an Unfavorable Cervix: Labor Induction with Prostaglandin or Oxytocin? A Retrospective Matched Case Study. *J Clin Med* **2024**, *13*, 3384, doi:10.3390/jcm13123384.
3. Mlodawski, J.; Mlodawska, M.; Plusajska, J.; Detka, K.; Bialek, K.; Swiercz, G. Repeatability and Reproducibility of Potential Ultrasonographic Bishop Score Parameters. *J Clin Med* **2023**, *12*, 4492, doi:10.3390/jcm12134492.
